# RNA-Seq transcriptomic analysis reveals gene expression profiles of acetic acid bacteria under high-acidity submerged industrial fermentation process

**DOI:** 10.3389/fmicb.2022.956729

**Published:** 2022-09-29

**Authors:** Haoran Yang, Yating He, Jing Liao, Xin Li, Junhong Zhang, Wolfgang Liebl, Fusheng Chen

**Affiliations:** ^1^Hubei International Scientific and Technological Cooperation Base of Traditional Fermented Foods, Huazhong Agricultural University, Wuhan, Hubei, China; ^2^College of Food Science and Technology, Huazhong Agricultural University, Wuhan, Hubei, China; ^3^Chair of Microbiology, Technical University of Munich, Freising, Germany; ^4^Jiangsu Hengshun Vinegar Industry Co., Ltd, Zhenjiang, Jiangsu, China

**Keywords:** acetic acid bacteria, industrial submerged fermentation, high acidity, RNA-seq transcriptome, acid resistance mechanisms

## Abstract

Acetic acid bacteria (AAB) are Gram-negative obligate aerobics in Acetobacteraceae family. Producing acetic acid and brewing vinegars are one of the most important industrial applications of AAB, attributed to their outstanding ability to tolerate the corresponding stresses. Several unique acid resistance (AR) mechanisms in AAB have been revealed previously. However, their overall AR strategies are still less-comprehensively clarified. Consequently, omics analysis was widely performed for a better understanding of this field. Among them, transcriptome has recently obtained more and more attention. However, most currently reported transcriptomic studies were conducted under lab conditions and even in low-acidity environment, which may be unable to completely reflect the conditions that AAB confront under industrialized vinegar-brewing processes. In this study, we performed an RNA-Seq transcriptomic analysis concerning AAB’s AR mechanisms during a continuous and periodical industrial submerged vinegar fermentation process, where a single AAB strain performed the fermentation and the acetic acid concentration fluctuated between ~8% and ~12%, the highest acidity as far we know for transcriptomic studies. Samples were directly taken from the initial (CK), mid, and final stages of the same period of the on-going fermentation. 16S rRNA sequence analysis indicated the participation of *Komagataeibacter europaeus* in the fermentation. Transcriptomic results demonstrated that more genes were downregulated than upregulated at both mid and final stages. Kyoto Encyclopedia of Genes and Genomes (KEGG) enrich analysis reflected that the upregulated genes mainly carried out tricarboxylic acid cycle and oxidative phosphorylation processes, probably implying a considerable role of acetic acid overoxidation in AR during fermentation. Besides, upregulation of riboflavin biosynthesis pathway and two NAD^+^-dependent succinate-semialdehyde dehydrogenase-coding genes suggested a critical role of succinate oxidation in AR. Meanwhile, downregulated genes were mainly ribosomal protein-coding ones, reflecting that the adverse impact on ribosomes initiates at the transcription level. However, it is ambiguous whether the downregulation is good for stress responding or it actually reflects the stress. Furthermore, we also assumed that the fermentation stages may have a greater effect on gene expression than acidity. Additionally, it is possible that some physiological alterations would affect the AR to a larger extent than changes in gene expression, which suggests the combination of molecular biology and physiology research will provide deeper insight into the AR mechanisms in AAB.

## Introduction

Acetic acid bacteria (AAB) are Gram-negative obligate aerobics in the family of Acetobacteraceae (Saichana et al., 2015). Their multiple membrane-bound dehydrogenases are capable of incompletely oxidizing a series of alcohols, sugars, and sugar alcohols to the corresponding aldehydes, ketones, and carboxylic acids, which is named as oxidative fermentation and has been extensively applied in industries (Adachi et al., 2003; Matsushita and Matsutani, 2016; Qin et al., 2022). Of these, the production of acetic acid from incomplete oxidation of ethanol is one of the most important features of AAB and has been used for vinegar production around the world (Gullo et al., 2014; Yang et al., 2022). Strains within species of *Acetobacter pasteurianus* and *Komagataeibacter europaeus* are most commonly used for vinegar brewing, attributed to their remarkable ability to produce as well as tolerate acetic acid (Gullo et al., 2014; Wang et al., 2015a). Under certain conditions, AAB strains are able to produce as high as 20% of acetic acid (Sokollek et al., 1998). However, acetic acid will cause the stress from several aspects (Trcek et al., 2015), while only 0.5% of acetic acid is sufficient to impose a lethal effect on many other microorganisms (Conner and Kotrola, 1995). Therefore, knowledge on the acid resistance (AR) mechanisms of AAB might be critical to reduce the harmful effect of acetic acid on the cells during the fermentation process and further to promote the relevant productivity. In addition, such extraordinary AR mechanisms of AAB that are able to tolerate high acidity are also attracting interests from researchers.

In recent decades, several unique mechanisms that contribute to AR in AAB have been revealed, which have been comprehensively reviewed and elucidated in details (Trcek et al., 2015; Wang et al., 2015a; Nakano and Ebisuya, 2016; Xia et al., 2017; Qiu et al., 2021; Yang et al., 2022). In brief, these mainly include the involvement of pyrroloquinoline quinone (PQQ)-dependent alcohol dehydrogenase (ADH) that also plays an indispensable role in producing acetic acid (Chinnawirotpisan et al., 2003; Trcek et al., 2006), overoxidation of acetic acid through a specialized tricarboxylic acid (TCA) cycle where succinyl-CoA:acetate CoA transferase (also known as AarC) functions as succinyl-CoA synthetase (Mullins et al., 2008), two acetic acid pumping-out systems that are either depended on ATP (Nakano et al., 2006) or proton motive force (PMF; Matsushita et al., 2005), contribution of molecular chaperons (Okamoto-Kainuma and Ishikawa, 2016), and alterations in the compositions of cell membrane (Trcek et al., 2007; Li et al., 2019). These AR-conferring mechanisms are most discussed and accepted by scientists at present (Yang et al., 2022). Recently, some new mechanisms that might be involved in AAB’s AR have also been proposed, such as repair of DNA damages under high concentration of acetic acid by protein UrvA (Zheng et al., 2018), 2-methylcitrate cycle (Yang et al., 2019), contribution of two-component signal transduction systems (Xia et al., 2016b, 2020), as well as toxin-antitoxin systems (Xia et al., 2019, 2021).

Unfortunately, the overall AR mechanisms in AAB are still yet to be clarified. It is unclear whether the acetic acid tolerance is resulted from the combination of different biological activities or only benefits from one or few critical but individual and specific processes. In order to obtain a whole view as well as a relatively better understanding of the AR mechanisms in AAB, scientists have carried out omics studies within this field. As a result, several genomes of AAB strains participating acetic acid fermentation were sequenced and analyzed (Azuma et al., 2009; Wang et al., 2015b; Xia et al., 2016a), while proteomic approaches were extensively exploited and have provided a number of important findings regarding AAB’s AR mechanisms, which have been reviewed and summarized previously (Nakano and Fukaya, 2008; Xia et al., 2017; Yang et al., 2022). Nowadays, being considered as the important bond linking genome and proteome, transcriptome has been obtaining more and more attentions from the researchers, leading to the reports of many relevant studies. For example, Sakurai et al. (2012) conducted a DNA microarray transcriptomic study which analyzed the gene expression patterns of *A. aceti* NBRC 14818 when the cells were oxidizing ethanol; Okamoto-Kainuma and Ishikawa (2016) carried out an RNA-Seq transcriptomic analysis of AR mechanisms in *A. pasteurianus* NBRC 3283, with special attention to the contribution of molecular chaperons; Yang et al. (2019) reported a transcriptome study concerning AR mechanisms in *A. pasteurianus* CGMCC 1.41 under acetic acid fermentation conditions, which proposed 2-methylcitrate cycle as a potential AR-conferring pathway; Xia et al. (2020) compared the gene expression profiles under high (7%) and low concentration (1%) of acetic acid conditions, focusing on the involvement of two-component signal transduction systems and toxin-antitoxin systems in AR of *A. pasteurianus* Ab3. Recently, Wang et al. (2021) performed a transcriptomic study concerning *K. europaeus* CGMCC 20445 in ethanol-oxidating environment, elucidating the contribution of several different AR mechanisms in *K. europaeus* at different stages of acetic acid fermentation.

**Figure 1:**
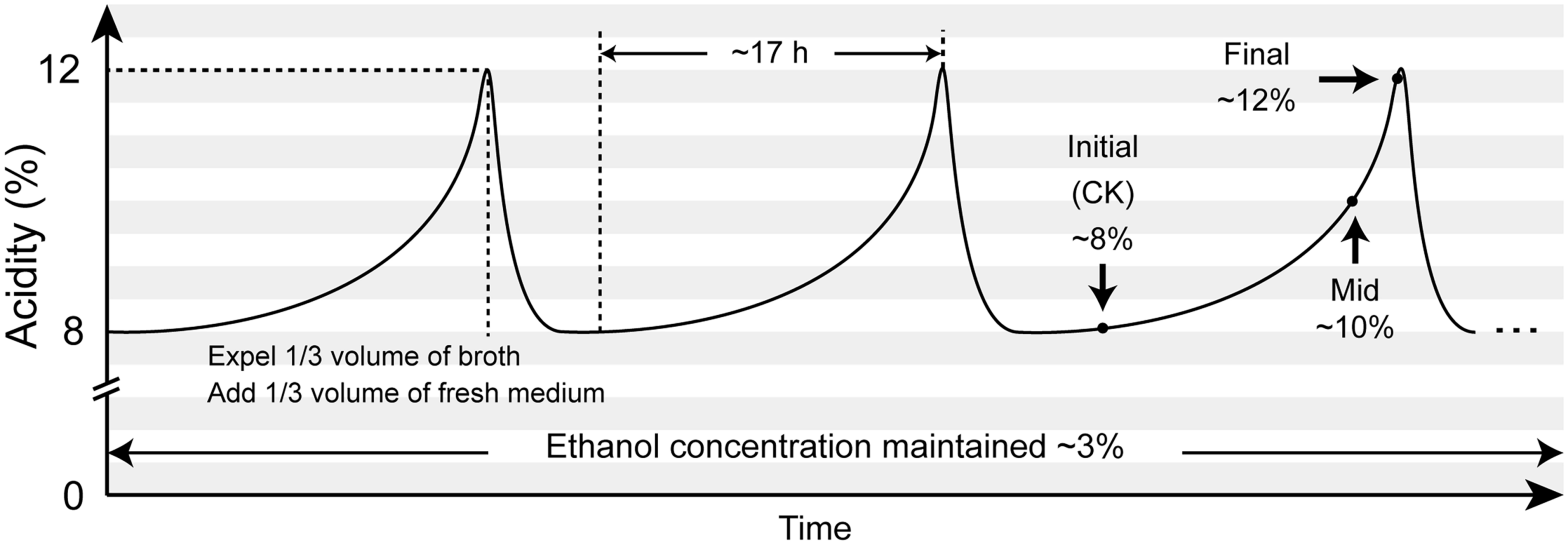
Illustration of the submerged liquid state fermentation process for sprit vinegar production and sampling plan. The submerged acetic acid fermentation was carried out uninterruptedly and periodically in a Chinese vinegar factory. Each fermentation period lasted for around 17 h, with the maximum acidity reaching around 12%. The concentration of ethanol was kept around 3% at all times. A fermentation period was terminated by discharging one-third volume of the fermentation broth, while a new period was initiated by adding another equal volume of the fresh medium. In this way, the acidity within the fermentor fluctuated between ~8% and ~12%. Samples for this transcriptomic study were collected at the initial, mid, and final stages within the same fermentation period.

However, these studies were all implemented under lab conditions, while some of these studies were even carried out in extremely low-acidity environment (around 1%). As AAB strains are commonly used for industrialized vinegar brewing process, inhabiting in the environment with much higher acidity than the lab conditions can provide, we believe that these studies may not be able to sufficiently reflect the genuine adversities that AAB strains have to deal with and therefore cannot provide the most valuable information for the industrial fermentation process. In this context, we intended to perform this RNA-Seq transcriptomic study within industrial background, collecting samples directly from an on-going continuous and periodical submerged liquid-state acetic acid fermentation process for the production of spirit vinegar in a Chinese vinegar factory, in which a single AAB strain performed the fermentation and the concentration of acetic acid fluctuated between ~8% and ~12% (Figure 1).

Here with this study, the analysis of gene expression patterns of AAB under this industrial fermentation as well as high acidity conditions will be presented, with a special focus on AAB’s AR mechanism. We took samples at the initial, mid, and final stages of the same fermentation period (Figure 1) and extracted total RNA for RNA-Seq transcriptome analysis. In parallel, a 16S rRNA sequence analysis together with the corresponding phylogenic analysis was also conducted in order to identify the AAB species executing the submerged fermentation and therefore select a proper reference genome for transcriptome analysis. Finally, a detailed elucidation of the transcriptome results based on Kyoto Encyclopedia of Genes and Genomes (KEGG) enrichment analysis would also be performed.

## Materials and methods

### Submerged fermentation, sample collection, and preparation

The submerged acetic acid fermentation was conducted uninterruptedly and periodically in the industrialized fermentor (Frings Co., Bonn, Germany). Bacterial strain used to execute the fermentation was provided by the fermentor manufacturer as part of the fermentation equipment. However, at the time of carrying out this study, the taxonomy background of this strain was unknown. Clarified and sterilized rice wine was constantly supplied in order to maintain the ethanol concentration around 3%. The fermentation was carried out at 30°C. When the acidity in the fermentor reached around 12%, which took about 17 h since a new fermentation period started, 1/3 volume of the fermentation broth was discharged for further processes of the spirit vinegar production. Subsequently, an equal volume of fresh medium prepared with Angel® Vinegar Fermentation Nutrient Salt (Angel Yeast Co., Ltd., Yichang, China) according to the instruction of the manufacturer was supplied to initiate a new period of fermentation, which simultaneously reduced the acidity in the fermentor to ~8% (Figure 1).

Samples were directly collected from the fermentor without disturbing the fermentation. At each scheduled sampling time point, that is, initial, mid, and final stages within the same fermentation period (Figure 1; Table 1), 50 ml of the sample was collected in a sterilized 50 ml centrifuge tube and was quickly put in ice, prior to which, 1 ml of the collected sample was pipetted out for determination of acidity by titration with 0.1 M NaOH solution. Cells in the remaining 49 ml sample were pelleted followed by washing twice with 25 ml ice-cold 1 M sodium acetate (pH 5.2). Finally, the washed cell pellets were suspended in 500 μl 1 M sodium acetate (pH 5.2) and stored under −80°C for 12 ~ 24 h prior to RNA extraction. The centrifuge was all carried out under 5,000 rpm for 10 min and 4°C for the above-mentioned manipulation. Two replicate samples were independently collected for each scheduled sampling point. Extraction of total RNA was performed as described by Yang et al. (2019).

**Table 1:** Sample information of the transcriptomic analysis.

Sample name	Acidity (%)	Time since the start of the fermentation period (h)
Initial (CK)	8.1	0.5
Mid	10.5	12.5
Final	11.7	16.5

### 16S rRNA sequence analysis

In order to identify the AAB species performing the fermentation, 16S rRNA fragment was amplified followed by sequence comparison and phylogenic analysis. This was carried out as described by Yang and Zhou (2014) with modification. 1 ml of the fermentation broth collected separately from the samples for RNA-Seq was centrifuged at 11,000 rpm for 2 min. The obtained pellet was then resuspended with 50 μl lysis buffer (Leagene Biotechnology Co., Beijing, China) and was put in a water bath of 95°C for 20 min, followed by centrifugation at 12,000 rpm for 2 min. The obtained supernatant was diluted by 10× with sterilized ddH_2_O and then was used as the template of PCR. PCR was conducted with Phanta Max Master Mix (Vazyme Biotechnology Co., Nanjing, China) in the volume of 25 μl and with primer pair P0 (5′-GAGAGTTTGATCCTGGCTCAG-3′) and P6 (5′-CTACGGCTACCTTGTTACGA-3′). PCR program was set according to the manual of PCR master mix. PCR products were then cleaned up with EasyPure PCR Purification Kit (TransGen Biotech Co., Beijing, China). Cleaned PCR products were subsequently the subjects for Sanger DNA sequencing, which was carried out by Sangon Biotech Co., Ltd. (Shanghai, China). The obtained sequence then acted as the query for conducting an online BLASTn search. The 16S rRNA sequence generated in this study now can be found at GeneBank^1^ with accession number OP164709.

For providing a clearer view of the taxonomy relationship, a phylogenic analysis based on 16S rRNA sequences was further conducted. Sequences from type strains of the relevant species were used to indicate the species position within the phylogenic tree. The information of species type strains was obtained from List of Prokaryotic names with Standing in Nomenclature.^2^ All 16S rRNA sequences except the one generated in this study were downloaded from NCBI Nucleotide database.^3^ Phylogenic tree was constructed by MEGA 7.0 software with the Neighbor-Joining method. Bootstrap method was used for phylogeny test with 1000 times of the bootstrap replications.

### Transcriptomic sequencing and data analysis

The purity and quality of the extracted RNA samples were evaluated by NanoDrop and Agilent 2100 Bioanalyzer, respectively. Upon good quality, rRNA was removed followed by the fragmentation of the samples. cDNA library was constructed with Illumina TruSeq Stranded Kit. Sequencing was then conducted with the HiSeq sequencing platform. The obtained raw reads were subsequently undergone a series of quality control processes including the removal of reads containing adaptor contamination as well as those containing more than 5% of the unknown bases (N) for downstream analysis. Reads with accepted quality were then mapped to the reference genome and reference genes with HISAT (2.0.1-beta; Kim et al., 2015) and Bowtie2 (2.2.5; Langmead and Salzberg, 2012) software, respectively. Based on the mapping data, RSEM (Li and Dewey, 2011) software was used to calculate the expression value of each gene, which was presented with Fragments Per Kilobase Million (FPKM) value. Based on the comparison scheme, differently expressed genes (DEGs) were identified by DESeq2 algorithm (Love et al., 2014) with the criteria of fold change ≥2 together with Bonferroni-corrected *p* value ≤0.05. The screened up- and downregulated genes of each treatment sample then separately acted as the subjects of KEGG enrichment analysis, which was carried out on OmicShare platform.^4^ KEGG terms with false discovery rate-corrected value of *p* ≤ 0.05 were regarded as the enriched terms of the up- or downregulated genes within a treatment sample. Expression data were visualized as heatmaps using TBtools software (Chen et al., 2020). The transcriptomic data now can be found at Gene Expression Omnibus database^5^ with accession number GSE210697.

## Results

### Microorganism identification, reference genome selection, and transcriptomic data overview

Since *K. europaeus* is regarded as the participator of submerged acetic acid fermentation (Gullo et al., 2014; Wang et al., 2015a), the strain inhabiting within the fermentor and conducting the fermentation in this study was expected to belong to this species. In order to verify this and therefore select a proper reference genome for transcriptome analysis, a 16S rRNA gene comparison was carried out prior to any other analysis. Online BLAST analysis results are listed in Table 2, which showed many hits to *Komagataeibacter* strains. Even though *Bacillus amyloliquefaciens* is also present in Table 2, we believe that this was an error in the information of the relevant sequence (MH824159), as the BLAST search using this sequence as the query only resulted in hit to *Komagataeibacter* 16S rRNA sequences instead of any other sequences from *Bacillus*, while currently there is also no report presenting the involvement of *Bacillus* species in submerged acetic acid fermentation. Meanwhile, *K. xylinus* and *K. swingsii* are also shown in Table 2. According to a phylogenic tree of 16S rRNA sequence from type strains of all AAB species reported by July 2021 (Yang et al., 2022), 16S rRNA sequence of *K. europaeus* and *K. swingsii* were identical, which explains the presence of *K. swingsii* in Table 2. In contrast, distance exists between *K. europaeus* and *K. xylinus*. On the other hand, *K. xylinus* strains shown in Table 2 were reported to possess the outstanding cellulose-producing ability (Ryngajłło et al., 2019; Du et al., 2020; Lu et al., 2020), which is the prominent feature of species *K. xylinus* acting as the model organism for studying bacterial cellulose production (Gullo et al., 2018). However, the production of bacterial cellulose is common among *Komagataeibacter* strains (Ryngajłło et al., 2019), including *K. europaeus*, in which cellulose-producing ability was also reported (Dubey et al., 2017). Thus, we assume that the *K. xylinus* strains listed in Table 2 may actually belong to *K. europaeus*.

**Table 2:** Top 10 hits of online BLASTn search using the sequence of 16S rRNA PCR product amplified from the samples in this study as the query.

Strain name	*E* value	Identity	Accession	Sequence type
*Komagataeibacter europaeus* DHBR3702	0	100%	MH845618.1	16S rRNA
*Bacillus amyloliquefaciens* BH072-1	0	100%	MH824159.1	16S rRNA
*Komagataeibacter xylinus* TJU-D2	0	100%	MH588135.1	16S rRNA
*Komagataeibacter europaeus* SRCM101446	0	100%	CP021467.1	complete genome
*Komagataeibacter xylinus* E25	0	100%	CP004360.1	complete genome
*Komagataeibacter europaeus* KGMA0119	0	100%	AB818453.1	16S rRNA
*Komagataeibacter xylinus* DSM 46603	0	100%	MZ435949.1	16S rRNA
*Komagataeibacter swingsii* JCM 17123	0	100%	NR_113400.1	16S rRNA
*Komagataeibacter europaeus* NBRC 3261	0	100%	AB680040.1	16S rRNA
*Komagataeibacter xylinus* OTG001	0	100%	MT730006.1	16S rRNA

**Figure 2:**
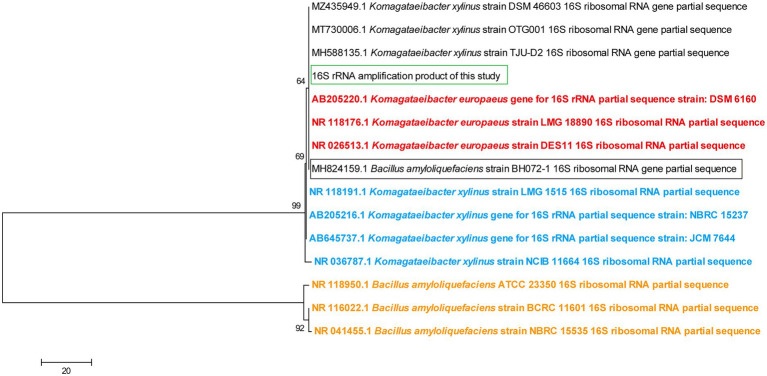
Neighbor-Joining phylogenic tree based on 16S rRNA sequences of relavant strains. Sequences from type strains of *Komagataeibacter europaeus*, *Komagataeibacter xylinus*, and *Bacillus amyloliquefaciens* were shown in red, blue, and orange font, respectively. 16S rRNA sequence that was amplified in this study and was from *B. amyloliquefaciens* BH072-1 was marked with a green and black box, respectively. Numbers next to the corresponding nodes indicate the bootstrap value (%) after 1,000 times of replication.

In order to further provide evidence for the hypotheses above, while given the fact that Table 2 could not provide a clear view of the taxonomy relationship, a Neighbor-Joining phylogenic tree was constructed using 16S rRNA sequences of the relevant strains, in which the sequences from the type strains of *K. xylinus*, *K. europaeus*, and *B. amyloliquefaciens* were set as the indicator of species position (Figure 2). It was demonstrated that the type strains of *B. amyloliquefaciens* and other *Komagataeibacter* species reside in two distinct clusters of the tree, while *B. amyloliquefaciens* BH072-1 stays with *K. europaeus* type strains, reflecting the error in taxonomy information about the sequence with accession number MH824159 on NCBI. Additionally, *K. xylinus* DSM 46603, OTG001, and TJU-D2, as well as 16S rRNA sequence generated in this study, were found present within the same subcluster as *K. europaeus* type strains, which indicated that these claimed *K. xylinus* strains may be more likely to belong to *K. europaeus* from this point of view, and that the genome of a *K. europaeus* strain can be a suitable reference genome for this study. As the fact that the complete genome of *K. europaeus* SRCM101446, which is also shown in Table 2 with 100% identity, was available at NCBI Genome data base (Bioproject No. PRJNA386757) at the time when this study was conducted, we therefore chose it as the reference genome for this transcriptome analysis.

**Figure 3:**
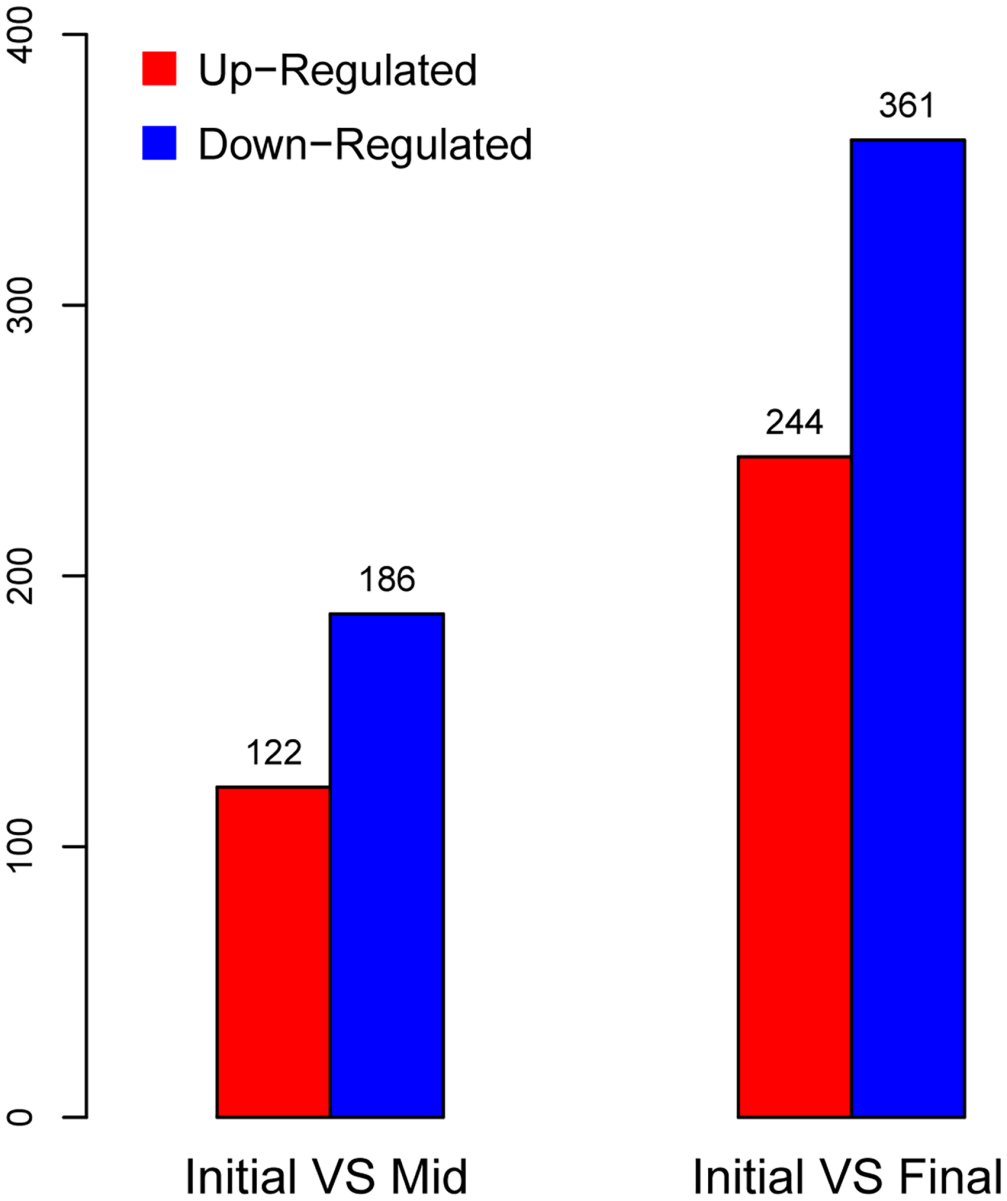
Statics of differently expressed genes (DEGs) within treatment samples.

Samples for this transcriptomic study were taken from the initial (acidity: 8.1%), mid (acidity: 10.5%), and final (acidity: 11.7%) stages of the same fermentation period of the continuous submerged liquid state fermentation process, in which the fermentation period had initiated for 0.5, 12.5, and 16.5 h, respectively (Figure 1; Table 1). Prior to cDNA library construction, the integrity of the extracted RNA was checked by Agilent 2100 Bioanalyzer and was regarded as qualified for further manipulation. In addition, reads obtained from RNA-Seq transcriptomic sequencing also passed the quality control, while more than 70% of the reads from each sample were mapped to the reference genome, which indicated that choosing the genome of *K. europaeus* SRCM101446 was feasible for this study and that the alignment results were sufficient to carry out subsequent expression qualification analysis. The sample group collected from the initial fermentation stage was set as the control group (CK) for the analysis regarding DEGs. Based on this, it was found that more genes were downregulated than upregulated when the fermentation entered mid and final stages, that is, 186, 361 genes were downregulated, while only 122,244 genes were upregulated at the mid and final stage of fermentation, respectively (Figure 3). In addition, it is also worth noting that the number of DEGs in the final stage was around twice as many as that in the mid stage, suggesting that cells were undergone more changes in biological activities in respond to the increasing amount of acetic acid.

In order to further elucidate the transcriptomic data and obtain more insight into the gene expression profile, the following paragraphs will present a detailed analysis based on KEGG enrichment analysis, of which the detailed results are shown in Supplementary Table S1.

### Detailed transcriptomic data analysis based on KEGG enrichment

#### TCA cycle and oxidative phosphorylation

**Figure 4:**
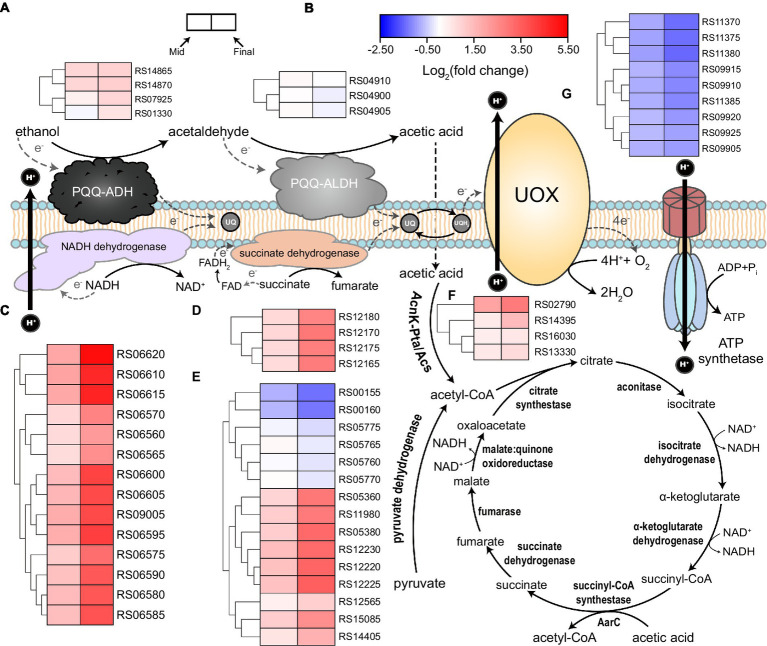
Illustration of respiratory chain and tricarboxylic acid (TCA) cycle together with the expression patterns of related genes. Acetic acid bacteria (AAB) cells possess both ethanol respiratory chain, which consists of PQQ-ADH and PQQ-ALDH, and common respiratory chain based on NADH and succinate dehydrogenase. Both respiratory chains deprive electrons from corresponding substrates and transfer them to ubiquinone (UQ), which is consequently reduced to ubiquinol (UQH_2_). UQH_2_ then transfers the electrons to ubiquinol oxidase (UOX) so that to be oxidized back to UQ. Finally, oxygen gains electrons from UOX, and then water is formed. NADH dehydrogenase and UOX are able to utilize the energy released from relevant redox reactions to expel protons out of the cells, generating proton motive force that can be used to yield ATP by ATP synthetase. Acetic acid produced by the ethanol respiratory chain from the incomplete oxidation of ethanol can penetrate into the cells and then is transferred to acetyl-CoA, which can be conducted either by the combination of acetate kinase (AckA) and phosphotransacetylase (Pta), or acetyl-CoA synthetase (Acs), or AarC. These processes enable acetic acid to enter TCA cycle and therefore act as a carbon source. Gene expression patterns (heatmap) together with the corresponding gene ID are present besides the relevant components or metabolic pathways, in which, **(A)** PQQ-ADH; **(B)** PQQ-ALDH; **(C)** NADH dehydrogenase; **(D)** succinate dehydrogenase; **(E)** TCA cycle; **(F)** AckA, Pta, and Acs; and **(G)** ATP synthetase. Detailed gene annotations and expression data are shown in Supplementary Table S2.

Tricarboxylic acid cycle and oxidative phosphorylation both play critical roles in energy metabolism, in which, a large amount of NADH is produced by TCA cycle and will be further oxidated through oxidative phosphorylation, that is, respiratory chain, to generate PMF for ATP synthesis. In the context of AAB’s acetic acid production and AR mechanisms, both of these processes have gained much attention, as TCA cycle plays a dominate part in acetic acid overoxidation, while enzymes that produce acetic acid from incomplete ethanol oxidation, that is, PQQ-ADH and PQQ-aldehyde dehydrogenase (ALDH; Saichana et al., 2015), also form a special ethanol respiratory chain (Yakushi and Matsushita, 2010; Yang et al., 2019, 2022; Figure 4). It was found that KEGG terms of “TCA cycle” and “oxidative phosphorylation” were significantly enriched within the upregulated genes in both mid and final stages of fermentation (Supplementary Table S1). As TCA cycle is regarded as the major pathway responsible for acetic acid overoxidation, while the subsequently generated NADH and succinate can participate in electron transfer process that yields a large amount of ATP, the significant upregulation of almost all the genes involved in both metabolic processes and the enrichment of relevant KEGG terms may reflect that acetic acid overoxidation probably still contributes considerably to AR in AAB under acetic acid fermentation conditions, especially in terms of the energy metabolism, even though previously it was not regarded as the main AR-conferring mechanism during ethanol oxidation (Kanchanarach et al., 2010; Yang et al., 2019). In addition, it was also demonstrated that *acs* (RS16030, RS02790), *ackA* (RS13330), and *pta* (RS14395), of which the product convert acetic acid into acetyl-CoA, were all upregulated during the fermentation process, while significant changes in the expression of these genes was not observed in a previous transcriptomic study regarding *A. pasteurianus* (Yang et al., 2019). Moreover, *K. europaeus* simultaneously harbors genes encoding common succinyl-CoA synthetase complex (RS00155 and RS00160) and AarC (RS05360), where the former were downregulated and the latter was upregulated (Figure 4; Supplementary Table S2). These together might indicate that *K. europaeus*, especially under industrial submerged fermentation conditions, can overoxidize acetic acid in a more effective way, which is therefore in line with the fact that *K. europaeus* is able to tolerate more acetic acid than *A. pasteurianus* (Wang et al., 2015a; Barja et al., 2016). However, both in mid and final stages, changes in the expression of almost all of the genes encoding the components of ethanol respiratory chain, that is, PQQ-ADH and PQQ-ALDH, did not meet the set criteria of DEGs (fold change ≥2, Figure 4; Supplementary Table S2), probably due to the constant concentration of ethanol (around 3%) throughout the fermentation process. On the other hand, the fold change of PQQ-ADH genes, including *adhB* (RS14865), *adhA* (RS14870), and its two paralogs (RS07925 and RS01330), was higher than those of PQQ-ALDH, with RS14870 was even found upregulated more than 2-fold at the final stage of the fermentation (Supplementary Table S2), which may again emphasize the contribution of PQQ-ADH to the AR when cells are facing high concentration of acetic acid. In addition, genes encoding ATP synthetase complex were found downregulated under higher acidity conditions (Figure 4; Supplementary Table S2), which was more likely to be resulted from the stress of a large amount of acetic acid that was imposed on the cells.

#### Riboflavin biosynthesis pathway and succinate-semialdehyde dehydrogenase

**Figure 5:**
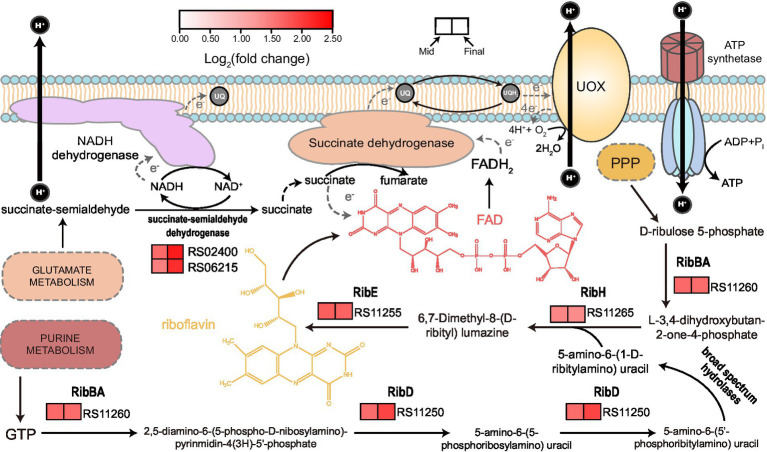
Illustration of riboflavin biosynthesis and its relationship to succinate oxidation together with relevant gene expression patterns. GTP from purine metabolism and D-ribulose 5-phosphate from pentose phosphate pathway (PPP) act as the start point of riboflavin biosynthesis. FAD is one of the derivatives of riboflavin and is related to succinate oxidation, which is a part of respiratory chain, as demonstrated in Figure 4. Succinate can be also obtained from oxidation of succinate-semialdehyde in an NAD^+^-dependent manner, which is also related to glutamate metabolism. Expression patterns (heatmap) with relevant gene ID are shown besides the biochemical reactions catalyzed by the corresponding gene products. Detailed expression data and gene annotations are listed in Supplementary Table S2. RibBA, 3,4-dihydroxy-2-butanone-4-phosphate synthase; RibD, bifunctional diaminohydroxyphosphoribosylaminopyrimidine deaminase; RibE, riboflavin synthase; and RibH, 6,7-dimethyl-8-ribityllumazine synthase.

“Riboflavin metabolism” is another KEGG term that was significantly enriched within the upregulated genes (Supplementary Table S1). Subsequently, we discovered that all the genes responsible for riboflavin biosynthesis were upregulated at both mid and final stages of fermentation (Figure 5). Riboflavin can act as the backbone of a variety of relevant derivatives (such as FMN and FAD) that consist of several flavoproteins, which are mainly involved in a series of redox biochemical reactions and photosensitization (García-Angulo, 2017). To our knowledge, currently, there is no report concerning the effect of light on *K. europaeus* and other AAB species. Hence, it is reasonable to assume that the upregulation of riboflavin biosynthesis pathway mainly contributed to redox processes, especially those related to energy metabolism, which might be a very important biological activity during later stages of acetic acid fermentation. Besides, KEGG term of “Butanoate metabolism” was also present in the enriched list within upregulated genes (Supplementary Table S1), where only genes coding for enzymes related to succinate metabolism were upregulated, including succinate dehydrogenase complex, AarC, and two NAD^+^-dependent succinate-semialdehyde dehydrogenases. Succinate-semialdehyde dehydrogenase catalyzes the oxidation of succinate-semialdehyde, which yields succinate and is involved in glutamate metabolism (Fuhrer et al., 2007). As it is well-known that succinate dehydrogenase complex, of which the relevant genes were also found upregulated in this study, operates in an FAD-dependent manner and therefore requires the support of riboflavin supply, these findings here together may suggest a critical role of succinate when AAB cells have to deal with the relatively high amount of acetic acid, in particular, under the context of succinate oxidation within the field of energy metabolism.

#### Downregulated ribosome-related protein-coding genes

**Figure 6:**
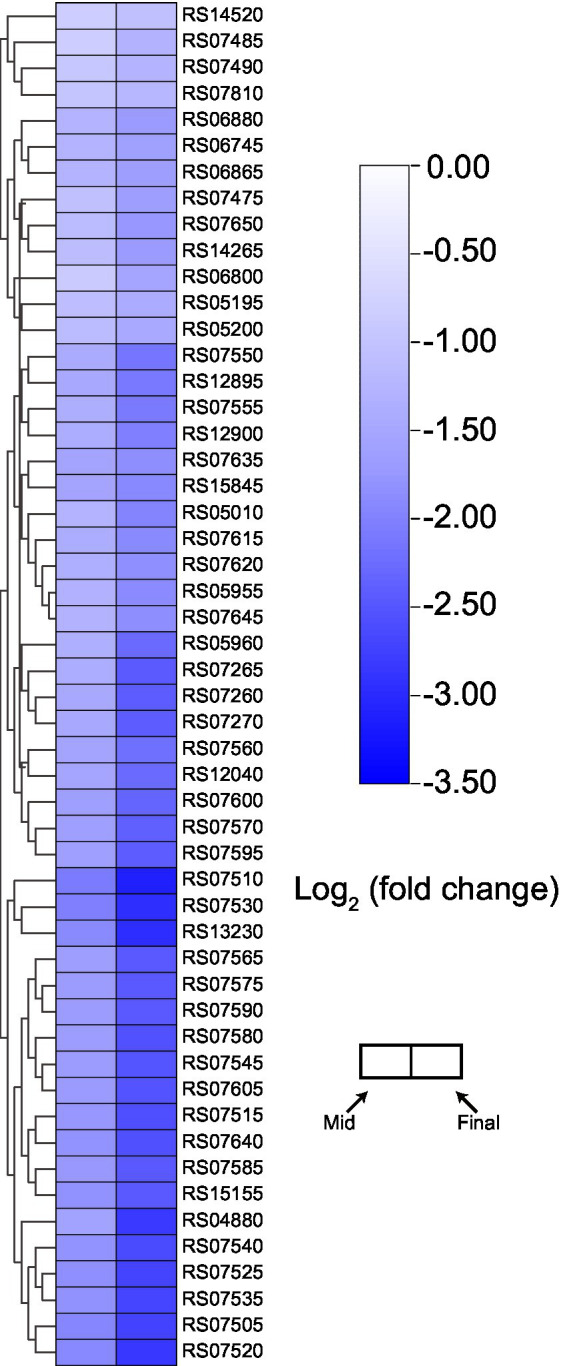
Expression pattern of genes encoding ribosome component. Detailed gene annotation and expression data are listed in Supplementary Table S2.

Among downregulated genes, there were almost no KEGG terms that were significantly enriched, except for “Ribosome” (Supplementary Table S1). Many ribosomal protein-coding genes were significantly downregulated during the mid and final stages of the fermentation (Figure 6). Previously, proteomic analysis indicated that ribosomal proteins would be downregulated as the concentration of acetic acid increases (Andrés-Barrao et al., 2012; Xia et al., 2016b). In addition, a recent transcriptomic study regarding *K. europaeus* also demonstrated that genes encoding ribosomal proteins were downregulated during the fermentation process (Wang et al., 2021). Hence, our results here once again suggest that the downregulation of the expression of ribosomal proteins may initiate at the transcription level. As their critical role in protein biosynthesis, ribosomes are always essential for all kinds of biological activities. Therefore, the downregulation of ribosome-related genes might actually reflect the stress from high concentration of acetic acid. However, at present, more evidence is still required to clarify the actual relationship between the downregulated ribosome-related genes and high acetic acid concentration.

Other downregulated genes were expected to involve in a wider range of different metabolic processes, as the fact that they accounted for the majority of DEGs but were rarely enriched to certain KEGG terms. Meanwhile, although more KEGG terms were enriched within the upregulated genes, it was hard to obtain clear clues from most of the enriched terms, as many upregulated genes within these terms cannot form intact presently known and curated metabolic pathways. Therefore, it is difficult to clarify the relationship between these genes and biological activities under acetic acid fermentation conditions at present, which requires further comprehensive and detailed studies.

## Discussion

In this study, we reported an RNA-Seq transcriptomic analysis of AAB concerning their AR mechanisms under acidity between ~8% and ~ 12%, the highest acidity as far we know for transcriptomic studies. Besides, our samples were directly taken from the production line of an industrial submerged acetic acid fermentation process, revealing gene expression patterns under realistic application background. Therefore, we believe the results presented in this study are of more practical value, which simultaneously can provide important evidence to support AR mechanisms that were proposed previously.

Our analysis reflected that the contribution of overoxidation of acetic acid to AR during fermentation may not be ignored, although the catabolism of acetic acid has been regarded as undesired and suppressed to some extent during acetic acid fermentation, since it is contradictory to the aim and the fact of the accumulation of a large amount of acetic acid (Kanchanarach et al., 2010; Yang et al., 2019). Besides, in a previous transcriptomic analysis, the shift of the main means of energy supply from ethanol oxidation to acetic acid overoxidation was presented, in which the degree of downregulation (compared to non-ethanol-oxidating conditions) of both TCA cycle and oxidative phosphorylation process based on NADH and succinate dehydrogenases was alleviated as the fermentation went by (Yang et al., 2019), a finding that was actually in accordance with the upregulation of both of these processes shown in this study. The trade-off between the common respiratory chain and the special respiratory chains for oxidative fermentation may extensively exist among more AAB species (Kostner et al., 2015). Unfortunately, here, we could not provide more direct evidence supporting this shift. This might be due to the reasons from two aspects. Firstly, in the aforementioned transcriptomic study, the control sample was collected from the culture without producing acetic acid, which was unable to be performed under industrial submerged fermentation conditions in this study, and consequently, we could not show the expression patterns of relevant genes that are exactly similar with previous one, in particular, drastic downregulation of TCA cycle and common respiratory chain at the initial stage of fermentation was absent. Secondly, significant downregulation of genes coding for ethanol respiratory chain was also not observed in this study (Figure 4). As the concentration of ethanol was always kept around 3% throughout the fermentation process, cells might use ethanol respiratory chain to constantly oxidize ethanol for energy generation. From this point of view, the balance effect between the two different respiratory chains and different means of energy metabolism may not be as obvious and necessary as under the conditions of previous studies. Furthermore, the expression of genes encoding the PQQ-ADH complex retained at a relatively high level during the whole fermentation period, while gene *adhA* was even up-regulated about 2-fold at the final stage (Figure 4; Supplementary Table S2), which probably suggested again the importance of PQQ-ADH in AR.

Succinate, the compound itself and/or the related metabolisms, may also play a considerable part in stress respond during acetic acid fermentation. In this study, genes coding for enzymes that participate in several processes with regard to succinate were found upregulated, including succinate-semialdehyde dehydrogenases (Figure 5), AarC, and succinate dehydrogenase complex (Figure 4). Both succinate-semialdehyde dehydrogenases and AarC catalyze reactions generating succinate, while succinate dehydrogenase is responsible for its oxidation. It is worth noting that succinate-semialdehyde dehydrogenases may also be associated with glutamate metabolism, while adding glutamate was found positively related to AR in *A. pasteurianus* (Yin et al., 2017). Hence, it is possible that the additional glutamate in the culture strengthened the AR partially by means of the enhanced succinate supply. In addition, adding succinate has been proven to be able to enhance acetic acid fermentation (Qi et al., 2013) while two previously proposed potential AR-conferring metabolic pathways in *Acetobacter* strains, that is, 2-methylcitrate cycle (Yang et al., 2019) and glyoxylate pathway (Sakurai et al., 2013), can also result in an additional supply of succinate. These findings altogether may further demonstrate the relationship between succinate and AR. Moreover, genes within the riboflavin biosynthesis pathway were significantly upregulated as well (Figure 5), which was assumed to link with succinate oxidation to some extent. Under such context, it is suggested that the involvement of succinate in AR of AAB may particularly lie in the contribution to the common respiratory chain, which was also found upregulated (Figure 4) and discussed above. Therefore, we believe that the further investigation into the relationship between succinate, perhaps also together with its oxidation metabolism, and AR may lead to more novel findings.

Although downregulated genes accounted for a large fraction of DEGs (Figure 3), almost all of their KEGG terms did not show significant enrichment, with the exception of “Ribosome” (Figure 6; Supplementary Table S1). The downregulation of ribosome-related genes under higher acidity conditions, both on transcription (Wang et al., 2021) and translation level (Andrés-Barrao et al., 2012; Xia et al., 2016b), was also demonstrated previously. Besides, genes coding for ATP synthetase complex were as well downregulated at the mid and final stages of fermentation (Figure 4), despite that the related KEGG terms were not significantly enriched within downregulated genes. The degree of downregulation of genes related to ATP synthetase and ribosome, which are both well-known to be vital to the basic biological activities of the cells, even increased at the final stage of fermentation (Figures 4, 6). However, it is incredible to draw such a conclusion that the downregulation of the essential genes is beneficial to the cells, or, these genes are harmful to the cells under stressful environment. On the other hand, since here we are quite in short of more compelling evidence, it is as well not easy to confirm that the downregulation itself actually reflected the stress, that is, the adverse conditions inhibit the expression of these genes, resulting in a number of negative effects on cells’ living activities, even though we would tend to accept this explanation. Do cells need actively downregulate these genes to cope with the stress, or, they are just unable to maintain the normal expression when encountering the stress? This is the question that all omics studies may need to handle, which, however, we pessimistically believe that none of the relevant omics studies can properly answer at the moment. In addition, although it is more rational to presume that cells would upregulate some genes and choose corresponding mechanisms to effectively deal with the stress, the upregulation may also instead act as the reflection of how the cells struggle within the stress, trying to seize the little opportunity for their survival. From this point of view, the relationship between DEGs and the stress might be more ambiguous and complicated than we expected and is hard to be clearly and objectively elucidated without further investigation. Consequently, we believe that researchers may need to be more cautious when discussing the effect of DEGs on stress responses, especially in the case of down-regulated genes. Besides, more comprehensive investigations are required to be involved to provide more experimental evidence for clarifying the relevant mechanisms in detail.

In this transcriptomic analysis, we obtained the expression patterns of many genes that were similar with the previous studies (Yang et al., 2019; Wang et al., 2021), even though the acidity we presented here was much higher. Nevertheless, it is worth noting that our sampling scheme was also similar with the previous ones, that is, we all planned to collect the samples at different stages of the fermentation. Thus, the combined changes of various factors within the environments of different fermentation stages are expected to have a greater impact on gene expression than acetic acid concentration alone. On the other hand, it is reasonable to expect that upon the survival of the cells, AR mechanisms of AAB work in a more effective way in high acidity environment than under low-acidity conditions. To this end, some physiological changes would be likely to affect the AR to a larger extent than alterations in gene expression, perhaps, such as the previously proposed but yet to be characterized PMF-dependent acetic acid pumping-out system (Matsushita et al., 2005). Additionally, AatA, an ATP-binding cassette transporter for acetic acid, was proved to be involved in AR in AAB (Nakano et al., 2006; Nakano and Fukaya, 2008). However, the transcription of gene *aatA* did not show a significant change as the acidity increased during fermentation (Sakurai et al., 2012; Yang et al., 2019), indicating that the contribution of AatA may not be reflected on the transcription level. In fact, the AR and acetic acid productivity of AAB has been regarded as unstable, as the relevant abilities might be immediately lost without genetical changes if the cells are grown in a acetic acid-free or non-ethanol-oxidating environment (Azuma et al., 2009), which may suggest that maintaining a specific physiological status is crucial to the capabilities of defending the stress from acetic acid. In this case, omics studies focusing on gene expression may have less chance to find out the key factor in AR mechanisms. As a result, researchers probably have to pay more attention to the investigation on physiological activities in terms of AAB’s AR mechanisms.

In general, we believe that something other than gene expression could make a more considerable difference in the AR mechanisms of AAB. Therefore, physiological analysis with regard to this field deserves more efforts from the scientists. Additionally, molecular biological research concentrating on the relevant genetic background is still one of the indispensable parts in terms of clarifying the underlying mechanisms. Hence, the combination of molecular biology and physiology research would be a better choice for related studies. Overall, we expect that by carrying out more well-designed and comprehensive studies, a deeper understanding and knowledge of the overall AR mechanism of AAB will be gained in the further.

## Data availability statement

The datasets presented in this study can be found in online repositories. The names of the repository/repositories and accession number(s) can be found in the article/Supplementary material.

## Author contributions

FC supervised the entire work and planned the experiments. HY carried out the majority of experiments, performed the analysis, and wrote the manuscript. YH and JL participated in the preparation of the figures. XL is in charge of the submerged fermentation and revised the manuscript. JZ contributed to 16S rRNA analysis. WL and FC revised the manuscript. All authors contributed to the article and approved the submitted version.

## Funding

This work was funded by Major Special Projects of Technological Innovation of Hubei Province, China (No. 2018ABA075), Programs of International S&T Cooperation, Ministry of Science and Technology, China (No. 2014DFG32380), and China Scholarship Council (202006760070).

## Conflict of interest

XL and JZ are employed by the company Jiangsu Hengshun Vinegar Industry Co., Ltd.

The remaining authors declare that the research was conducted in the absence of any commercial or financial relationships that could be construed as a potential conflict of interest.

## Publisher’s note

All claims expressed in this article are solely those of the authors and do not necessarily represent those of their affiliated organizations, or those of the publisher, the editors and the reviewers. Any product that may be evaluated in this article, or claim that may be made by its manufacturer, is not guaranteed or endorsed by the publisher.

## Footnotes

^1^ https://www.ncbi.nlm.nih.gov/genbank/

^2^ https://lpsn.dsmz.de/

^3^ https://www.ncbi.nlm.nih.gov/nuccore/

^4^ http://www.omicshare.com/tools/

^5^ https://www.ncbi.nlm.nih.gov/geo/
